# Complete Genome Sequences of Two *Pseudomonas* Species Isolated from Marine Environments of the Pacific Ocean

**DOI:** 10.1128/MRA.01062-19

**Published:** 2021-04-22

**Authors:** Shi-Zhen Wang, Corinne Cruaud, Jean-Marc Aury, David Vallenet, Julie Poulain, Benoit Vacherie, Anne Zaparucha, Carine Vergne-Vaxelaire

**Affiliations:** a Department of Chemical and Biochemical Engineering, Xiamen University, Xiamen, China; b Genoscope, Institut de Biologie François Jacob, CEA, Université Paris-Saclay, Evry, France; c Génomique Métabolique, Genoscope, Institut François Jacob, CEA, CNRS, Univ Evry, Université Paris-Saclay, Evry, France; University of Arizona

## Abstract

Pseudomonas marincola YsY11 and Pseudomonas oleovorans T9AD were both isolated from marine environments of the Pacific Ocean. Here, we report the whole-genome sequences of these two organisms. Pseudomonas marincola YsY11 consists of a single 4.77-Mb chromosome, and Pseudomonas oleovorans T9AD consists of a 5.57-Mb chromosome and a 2.8-kb plasmid.

## ANNOUNCEMENT

Water environments, including oceans, represent an immense reservoir of unknown organisms. Various samplings have been carried out to start obtaining an overview of the organisms present in these ecosystems. Pseudomonas marincola YsY11 and Pseudomonas oleovorans T9AD were obtained from the Marine Culture Collection of China (MCCC). They were both isolated from marine environments of the Pacific Ocean. Both strains were received in two freeze-drying storage tubes. One tube was frozen as our own stock, and the other one was started for isolation.

Pseudomonas marincola is an aerobic, Gram-negative, motile bacterium. Pseudomonas oleovorans is a Gram-negative, methylotrophic bacterium that can produce poly(3-hydroxyl alkanoate) (1). Pseudomonas marincola YsY11 and Pseudomonas oleovorans T9AD were cultured in marine broth 2216 medium, for which the optimal growth temperature was found to be 35°C. A single colony was picked for liquid cultivation for both strains. From a culture at 30°C, genomic DNA was purified using the ZR-Duet DNA/RNA MiniPrep Plus kit (D7003; Zymo Research). Genome sequencing was performed at Genoscope, using both Illumina (PCR-free library) and Oxford Nanopore Technologies (SQK-LSK108 protocol) systems, from 2,562 ng and 9,630 ng genomic DNA from Pseudomonas marincola YsY11 and Pseudomonas oleovorans T9AD, respectively (Table 1).

**TABLE 1:** Statistics for the Oxford Nanopore Technologies and Illumina data sets

Parameter	Data for Pseudomonas marincola YsY11		Data for Pseudomonas oleovorans T9AD	
	ONT^*a*^	Illumina	ONT	Illumina
No. of reads	1,432,208	2,564,800	1,033,583	2,348,875
Cumulative size (bp)	6,812,095,470	1,228,989,938	7,533,926,993	1,127,246,391
Avg size (bp)	4,756	251	7,289	251
*N*_50_ (bp)	12,649	14,973
Maximum size (bp)	1,141,486	251	500,202	251
Coverage (×)	1,416	255	1,341	200
SRA accession no.	ERX3585526	ERX3585525	ERX3585527	ERX3585528

a ONT, Oxford Nanopore Technologies.

The genomes of both strains were assembled using the same strategy. Default parameters were used unless otherwise specified. First, a subset of 30× of the long reads generated by the MinION system were selected using Filtlong software (https://github.com/rrwick/Filtlong). These reads were assembled using Flye (version 2.8) (2). The resulting assembly was then polished using Racon (3) (with all of the long reads) and two rounds of Hapo-G (4) with the help of 2 × 251-bp paired-end reads generated by a MiSeq sequencer with genome coverage of ∼200×. The Pseudomonas marincola YsY11 assembly was composed of a single contig of 4.77 Mb, with a GC content of 57.3%; the Pseudomonas oleovorans T9AD assembly was composed of two contigs of 5.57 Mb and 2.8 kb, with GC contents of 64.5% and 61.8%, respectively.

Automatic functional annotation was performed using the MicroScope platform (5) and submitted to the ENA databank along with the genome sequences. In total, 4,630 genomic objects were identified in Pseudomonas marincola YsY11, including 4,504 coding sequences (CDSs), 65 tRNAs, 16 rRNAs, and 44 noncoding RNAs. In Pseudomonas oleovorans T9AD, 5,583 genomic objects were identified on the chromosome, including 5,444 CDSs, 65 tRNAs, 12 rRNAs, and 61 noncoding RNAs. Only 4 CDSs were identified on the plasmid.

### Data availability.

The raw data are available from the EBI ENA database under the accession number PRJEB34870. The assembled genome sequences and their annotations were deposited in the ENA under the accession numbers LR215729, LR130779, and LR130780.
